# BSAlign: A Library for Nucleotide Sequence Alignment

**DOI:** 10.1093/gpbjnl/qzae025

**Published:** 2024-03-14

**Authors:** Haojing Shao, Jue Ruan

**Affiliations:** Shenzhen Branch, Guangdong Laboratory of Lingnan Modern Agriculture, Genome Analysis Laboratory of the Ministry of Agriculture and Rural Affairs, Agricultural Genomics Institute at Shenzhen, Chinese Academy of Agricultural Sciences, Shenzhen 518120, China; Shenzhen Branch, Guangdong Laboratory of Lingnan Modern Agriculture, Genome Analysis Laboratory of the Ministry of Agriculture and Rural Affairs, Agricultural Genomics Institute at Shenzhen, Chinese Academy of Agricultural Sciences, Shenzhen 518120, China

**Keywords:** Pairwise alignment, Edit distance, Striped vectorization, Banded dynamic programming, F evaluation

## Abstract

Increasing the accuracy of the nucleotide sequence alignment is an essential issue in genomics research. Although classic dynamic programming (DP) algorithms (*e.g.*, Smith–Waterman and Needleman–Wunsch) guarantee to produce the optimal result, their time complexity hinders the application of large-scale sequence alignment. Many optimization efforts that aim to accelerate the alignment process generally come from three perspectives: redesigning data structures [*e.g.*, diagonal or striped Single Instruction Multiple Data (SIMD) implementations], increasing the number of parallelisms in SIMD operations (*e.g.*, difference recurrence relation), or reducing search space (*e.g.*, banded DP). However, no methods combine all these three aspects to build an ultra-fast algorithm. In this study, we developed a Banded Striped Aligner (BSAlign) library that delivers accurate alignment results at an ultra-fast speed by knitting a series of novel methods together to take advantage of all of the aforementioned three perspectives with highlights such as active F-loop in striped vectorization and striped move in banded DP. We applied our new acceleration design on both regular and edit distance pairwise alignment. BSAlign achieved 2-fold speed-up than other SIMD-based implementations for regular pairwise alignment, and 1.5-fold to 4-fold speed-up in edit distance-based implementations for long reads. BSAlign is implemented in C programing language and is available at https://github.com/ruanjue/bsalign.

## Introduction

Nucleotide sequence alignment is a way to arrange and compare DNA/RNA sequences from different sources to identify their regions of similarity. Two classic algorithms, namely Needleman–Wunsch algorithm [1] and Smith–Waterman algorithm [2], are commonly used for sequence alignment. They handle sequence alignment by solving a dynamic programming (DP) problem in which a scoring matrix is calculated and an optimal path from the cell with a maximal score is returned. Although these two methods have shown high capability in finding optimal alignment results, they require quadratic time complexity and rapidly degenerate especially when processing long sequences. To accelerate the alignment process, three major categories of optimization techniques have been developed along the way.

### Single Instruction Multiple Data

The first optimization category is to redesign the data structure of the scoring matrix calculation to resolve data dependencies between neighboring cells so that the conditional branch within the inner loop of the DP algorithm can be eliminated and hence more efficient in parallelization techniques such as Single Instruction Multiple Data (SIMD). Among the initial trials in this category, Wozniak [3] has presented an implementation to store values parallel to the minor diagonal to eliminate the conditional branch in the inner loop of traditional implementation and achieved a 2-fold speed-up. In a different trial, Rognes et al. [4] introduced another implementation to store values parallel to the query sequences. Compared to Wozniak’s implementation, an advantage of Rognes’s design is that it only needs to compute the query profile once for the entire reference sequences. However, the disadvantage is that conditional branches are placed in the inner loop when evaluating F matrix. The length of a single instruction ranges from 128-bit to 512-bit for recent tools such as BGSA [5], SeqAn [6], and AnySeq [7].

### Striped SIMD and F evaluation

To combine the merits of both Wozniak [3] and Rognes et al. [4], Farrar [8] fixed these disadvantages by introducing a layout of query sequences that are parallel to the SIMD registers but are accessed in a striped pattern, which only computes query profile once and moves the conditional F matrix evaluation outside of the inner loop. As a result, Farrar’s striped vectorization successfully speeds up the Smith–Waterman algorithm and has been adopted by many aligners, such as Burrows–Wheeler Alignment Smith–Waterman (BWA-SW) [9], Bowtie2 [10], and Striped Smith–Waterman (SSW) library [11]. However, cells in the same register are not always independent of each other. Farrar [8] solved this problem by adding a correction loop for every F element, which may iterate many times when the insertions and deletions (indels) are long enough.

### Difference recurrence relation

The next optimization category is to increase the number of parallelisms in SIMD operations such as difference recurrence relation [12]. Since the traditional pairwise alignment stores and calculates the absolute values in the score matrix, it limits the width of SIMD operation as the sequence length increases. The difference recurrence relation solves this problem by only storing and calculating the differences between the adjacent cells, which keeps the full width of SIMD operation regardless of the sequence length. For example, the number of bits for storing the absolute value of a single cell is 16 or even 32. However, it can reduce to 8 bits if just storing the differences between cells. Therefore, the number of parallelisms increases by 2 to 4 times.

### Banded DP

Another optimization category is reducing the search space such as banded DP. Instead of calculating the whole score matrix, banded DP maintains a hypothetical “band” around cells with maximal scores, calculates the scores for cells within the “band” only, and skips calculating the remaining cells within the matrix [13,14]. How to combine the method of using SIMD (minor diagonal or striped) and the idea of reducing the search space is not clear, especially when the input sequences contain abundant indel errors by third-generation sequencers. Suzuki et al. [14] proposed a minor diagonal SIMD adaptive banded DP algorithm, which is implemented and improved in a popular long-read mapper minimap2 [15]. Since the striped SIMD method [8] was proved to be 6 times faster than the minor diagonal and other SIMD methods [3] in Smith–Waterman algorithm without banded DP, the algorithm to combine the best SIMD method with banded DP is not developed yet.

### Block aligner and wavefront algorithm

Recent methods block aligner (BA) [16] and wavefront algorithm (WFA) [17] manage to reduce the search space around the diagonal by two innovative approaches. BA starts the alignment by a small square block and extends the block dynamically until the endpoint is reached. The block could be shifted either down or right according to the sum of the cells. The size of the extended block may double when a Y-drop condition is met. The width of the block (band) depends on the sequence’s identity. Unlike the BA, the WFA regards the global alignment as the wave spreading from the start point to the endpoint. WFA extends the wave step-by-step until the endpoint is reached. To speed up, WFA utilizes the homologous region between the sequences to skip the path (wavefront) that is unlikely to lead to the optimal solution. The search space for the wavefront aligner is wave-like banding along the diagonal.

Overall, we presented a new library with an aligner, Banded Striped Aligner (BSAlign) library, which is able to combine merits from the aforementioned optimization techniques without bringing their respective limitations. Firstly, we developed an active F loop evaluation algorithm in the striped vectorization [8] to reduce the redundant F matrix recalculation, which accelerates the evaluation of the scoring matrix. We also introduced difference recurrence relation and developed a banded DP striped move algorithm to efficiently combine the striped SIMD method and banded DP. Finally, we designed a fast bit-vector algorithm to further speed up edit distance-based alignment.

## Method

### Overview

We developed a set of new methods to address the pairwise sequence alignment problem by adopting advantages from previous work like striped vectorization [8], difference recurrence relation [12], banded DP [13], and by proposing novel improvements like a technique called active F-loop evaluation, a set of newly derived recurrence relations, a variant of bit conversion for edit distance alignment (File S1), and different levels of adjustments to integrate all the features into the BSAlign.

### The striped move algorithm for banded DP

**Figure 1:**
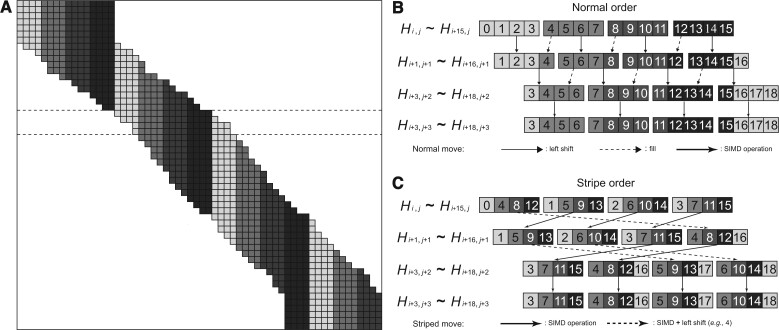
The striped move algorithm for each row **A**. Global visualization for the band along the diagonal. **B**. Detail example for row iteration in normal order. **C**. Detail example for row iteration in striped order (striped move). Assuming the band width, the number of divided segments, and the number of cells in a register are 16, 4, and 4, respectively. In normal order, the cells are in the same color for the same register. Only the offset is numbered inside the cell. SIMD, Single Instruction Multiple Data.

In the beginning, the algorithm calculates the global alignment by the Needleman–Wunsch algorithm [1] using striped SIMD [8] data structure (**Figure 1**A; File S1), which optimizes the pairwise alignment by focusing only on the alignment along a diagonal band. A difficulty in applying banded DP to the striped SIMD method is that the entire striped SIMD data structure rearranges each time the band moves along the diagonal (Figure 1B). To overcome this difficulty, we develop a method to move the striped SIMD data structure for banded DP (Figure 1C). In normal coordinate, the whole register **N** stores (Figure 1B):

(1) $$N_{0,j}=\left[H_{i\;+\;0,j},H_{i\;+\;1,j},H_{i\;+\;2,j},\ldots ,H_{i\;+\;p\;-\;1,j}\right]\;N_{1,j}=[H_{i\;+\;p\;+\;0,j},H_{i\;+\;p\;+\;1,j},H_{i\;+\;p\;+\;2,j},\ldots ,H_{i\;+\;2p\;-\;1,j}]\ldots N_{\bar{s}\;-\;1,j}=[H_{i\;+\;\left(\bar{s}\;-\;1\right)\;*\;p\;+\;0,j},H_{i\;+\;\left(\bar{s}\;-\;1\right)\;*\;p\;+\;1,j},H_{i\;+\;\left(\bar{s}\;-\;1\right)\;*\;p\;+\;2,j},\ldots ,H_{i\;+\;\left(\bar{s}\;-\;1\right)\;*\;p\;+\;p\;-\;1,j}]$$

where *i* and *j* are the first column and row coordinate in the memory; *H* represents the alignment score; *p* represents the number of cells; and $\bar{s}$ is the band width divided by *p*. In the banded DP, the movement of the current row indicates the selection of the optimal path. Moving zero, one, and two cells to the right indicates one vertical gap, no gap, and one horizontal gap, respectively (Figure 1A). In our banded DP algorithm, we compare the *H* values in the first register and last register, move the current row zero, one, or two cells to the right according to the comparison, and prepare for the next row. In the next row, the start position $H_{i\;+\;1,j\;+\;1}$ depends on the current row $\left[H_{i,j},\ldots ,H_{i\;+\;\bar{s}\;*\;p\;-\;1,j}\right]$ as following:

(2) $$H_{i\;+\;x,j\;+\;1}=\left\{\begin{array}{cc} H_{i,j\;+\;1} & \left(\mathrm{Sum}\left(N_{0,j}\right)>\mathrm{Sum}(N_{\bar{s}\;-\;1,j})\right)\\ H_{i\;+\;1,j\;+\;1} & \left(\mathrm{Sum}\left(N_{0,j}\right)==\mathrm{Sum}(N_{\bar{s}\;-\;1,j})\right)\\ H_{i\;+\;2,j\;+\;1} & \left(\mathrm{Sum}\left(N_{0,j}\right)<\mathrm{Sum}(N_{\bar{s}\;-\;1,j})\right) \end{array}\right\}$$

$H_{i\;+\;x,j\;+\;1}$ move zero, one, and two cells to the right, which are showed as rows *j* + 3, *j* + 1, and *j* + 2 in Figure 1B and Figure 1C, respectively. We develop our striped move following the aforementioned Equations 1 and 2. In striped coordinates, the whole register **M** stores:

(3) $$M_{0,j}=\left[H_{i,j},H_{i\;+\;\bar{s},j},H_{i\;+\;2\;*\;\bar{s},j},\ldots ,H_{i\;+\;\left(p\;-\;1\right)\;*\;\bar{s},j}\right]M_{1,j}=[H_{i\;+\;1,j},H_{i\;+\;1\;+\;\bar{s},j},H_{i\;+\;1\;+\;2\;*\;\bar{s},j},\ldots ,H_{i\;+\;1\;+\;\left(p\;-\;1\right)\;*\;\bar{s},j}]\ldots \;M_{\bar{s}\;-\;1,j}=[H_{i\;+\;\bar{s}\;-\;1,j},H_{i\;+\;\bar{s}\;-\;1\;+\;\bar{s},j},H_{i\;+\;\bar{s}\;-\;1\;+\;2\;*\;\bar{s},j},\ldots ,H_{i\;+\;\bar{s}\;-\;1\;+\;\left(p\;-\;1\right)\;*\;\bar{s},j}]$$

The memory stores all the registers as $\left[M_{0,j},M_{1,j},M_{2,j},\ldots ,M_{\bar{s}-1,j}\right]$. In the striped order, the cell order for the first register in the *j* + 1 row (such as [*H*_1,*j* + 1_, *H*_5,*j* + 1_, *H*_9,*j* + 1_, *H*_13,*j *+ 1_]) is the same as the first, second, and third registers in the *j* row (such as [*H*_1,*j*_, *H*_5,*j*_, *H*_9,*j*_, *H*_13,*j*_]) for moving zero, one, or two cells to the right, respectively (Figure 1C). This holds true for all the registers except the last one or two registers. For the calculation of the next row, the memory is the following:

(4) $$\mathrm{Memory}=\left\{\begin{array}{cc} \left[M_{0,j},M_{1,j},M_{2,j},\ldots ,M_{\bar{s}\;-\;1,j}\right] & \left(\mathrm{Sum}\left(M_{0,j}\right)>\mathrm{Sum}(M_{\bar{s}\;-\;1,j})\right)\\ \left[M_{1,j},M_{2,j},\ldots ,M_{\bar{s}\;-\;1,j},M_{0}\right] & \left(\mathrm{Sum}\left(M_{0,j}\right)==\mathrm{Sum}(M_{\bar{s}\;-\;1,j})\right)\\ \left[M_{2,j},\ldots ,M_{\bar{s}\;-\;1,j}{,M}_{0,j},M_{1,j}\right] & \left(\mathrm{Sum}\left(M_{0,j}\right)<\mathrm{Sum}(M_{\bar{s}\;-\;1,j})\right) \end{array}\right\}$$

For the exception, the new $\bar{M}$ can be converted from the previous **M** (dash arrow in Figure 1C, such as [*H*_1,*j *+ 1_, *H*_5,*j* + 1_, *H*_9,*j* + 1_, *H*_13,*j* + 1_] to [*H*_5,*j* + 2_, *H*_9,*j* + 2_, *H*_13,*j* + 2_, *H*_17,*j *+ 2_]). Take ${\bar{M}}_{0,j+1}$ as an example:

(5) $${\bar{M}}_{0,j}=\left(M_{0,j}\ll l_{\mathrm{byte}}\right)+[0,0,0,\ldots ,-\infty ]$$

*l_byte_* is the byte length of *H*. As the row moves one cell to the right, the whole cells move from $\left[H_{i,j},\ldots ,H_{i\;+\;\bar{s}\;*\;p\;-\;1,j}\right]$ to $\left[H_{i\;+\;1,j\;+\;1},\ldots ,H_{i\;+\;\bar{s}\;*\;p,j\;+\;1}\right]$. The addition cell $H_{i\;+\;\bar{s}\;*\;p,j\;+\;1}\;$is set as the negative infinity and filled in the register. The negative infinity indicates that this boundary cell will not be selected by Equation 7. The banded algorithm skips the calculation of boundary cells to speed up the global alignment. Using our striped move method, the whole striped SIMD data only need bit operations to prepare for a new row in banded DP.

### Difference recurrence relation

The third way to optimize the pairwise alignment is to increase the number of parallelisms (*i.e.*, vector width) in SIMD operations. We choose to calculate the score matrix based on the difference recurrence relation [12] instead of the conventional stripped SIMD implementation [8]. We denote **h**, **e**, and **f** as the relative score of the **H**, **E**, and **F** score matrices, respectively. We also define **u** matrix and **v** matrix to represent the vertical difference and horizontal difference within the **H** matrix, respectively.

(6) $$\left\{\begin{array}{c} h_{i,j}=H_{i,j}\;-\;H_{i\;-\;1,j\;-\;1}\\ u_{i,j}=H_{i,j}\;-\;H_{i\;-\;1,j}=h_{i,j}\;-\;v_{i\;-\;1,j}\\ \begin{array}{c} v_{i,j}=H_{i,j}\;-\;H_{i,j\;-\;1}=h_{i,j}\;-\;u_{i,j\;-\;1}\\ e_{i,j}=E_{i,j}\;-\;H_{i,j\;-\;1}\\ f_{i,j}=F_{i,j}\;-\;H_{i\;-\;1,j\;-\;1} \end{array} \end{array}\right\}$$

The definition of **e** and **f** matrices is asymmetry. Under the aforementioned definition, our difference recurrence relation can be expressed as:

(7) $$\left\{\begin{array}{c} h_{i,j}=\max(S_{i,j}{,e}_{i,j}\;+\;u_{i,j\;-\;1},f_{i,j})\\ e_{i,j\;+\;1}=\max(e_{i,j}\;+\;u_{i,j\;-\;1}\;-\;h_{i,j}\;+\;\mathrm{GapE},\mathrm{GapOE})\\ f_{i\;+\;1,j}=\max\left(f_{i,j}\;+\;\mathrm{GapE},h_{i,j}\;+\;\mathrm{GapOE}\right)\;-\;u_{i,j\;-\;1} \end{array}\right\}$$

For every iteration in our implementation, we only store *u*_*i*,*j* − 1_ and *e*_*i*,*j*_, and calculate *h*_*i*,*j*_, *v*_*i*,*j*_, *e*_*i*,*j* + 1_, and *f*_*i* + 1,*j*_ by Equations 6 and 7.

### Active F loop

**Figure 2:**
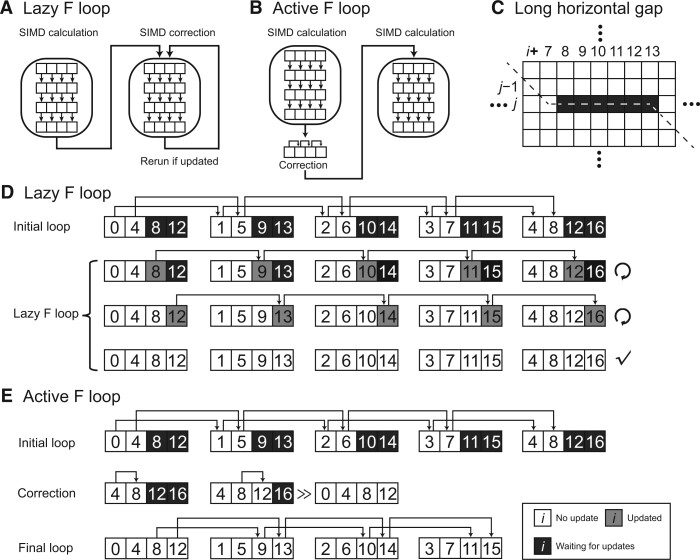
The lazy and active F loop algorithms inside a row Assuming the band width, the number of divided segments, and the number of cells in a register are 16, 4, and 4, respectively. **A**. and **B**. The workflow for the lazy (A) and active (B) F loops, respectively. **C**. An example of long horizontal gap (*f*_7,*j*_ to *f*_13,*j*_). The dash line indicates the optimal path. **D**. A diagram showing how the lazy F loop solves the aforementioned example for each cell. Initial loop: all the cells in the first register are negative infinity, and the algorithm calculates all the cells by standard SIMD calculation. Lazy F loop: the algorithm keeps correcting all the registers one by one until none of them is updated, and this panel shows a situation that it takes 3 loops to guarantee that all the cells are corrected. **E**. A diagram showing how the active F loop solves the aforementioned example for each cell. Unlike the lazy F loop, there is no difference between “updated” and “no update”. All the values are updated one times only. Initial loop: the same as the lazy F loop. Correction: each cell in the first segment is checked by Equation 10 and updated by the correct value. This extended register is also the first register (after the right shift) in the striped format. Final loop: when the first register is totally correct, the remaining segments are corrected by SIMD calculation. Black box (waiting for updates) indicates that the value is influenced by F and needs to be corrected. Gray box (updated) indicates that the value is updated recently. White box (no update) indicates that the value is the same as expected and correct. The arrows above digits indicate the first time being updated as the correct value.

For non-striped pairwise alignment, the horizontal score *F* and its difference *f* can be calculated step-by-step via previous cells. In striped order, some cells show up before their previous cells ([*f*_4_, *f*_8_, *f*_12_] in **Figure 2**A–E). Thus, it raises a problem only in calculating the horizontal score *F* and its difference *f*. Farrar [8] initially developed a lazy *F* evaluation method to solve this problem (Figure 2A). It corrected the value of *F* via a couple of loops, which is time-consuming for sequences that contain long indel errors (Figure 2D). In contrast to lazy *F* evaluation, we actively correct all the cells in advance and guarantee that the value of *H* is always corrected, providing a linear complexity solution to this problem in any situation (Figure 2B and E).

For most registers in the memory, *f* is smaller than *e* and *S*. The value of *H* does not source from *f* (Figure S1A). Only when there is the horizontal gap will *f* start to influence the value of *H* (Figure S1B and C). In the initial loop, we set the negative infinity as the first register **MF**_0,*j*_ for horizontal difference *f* ([*f*_0,_*f*_4,_*f*_8,_*f*_12_] in Figure 2E):

(8) $${\mathrm{MF}}_{0,j}=\left[f_{0,j},f_{\bar{s},j},f_{\bar{s}\;*\;2,j},\ldots ,f_{\bar{s}\;*\;(p\;-\;1),j}\right]$$

Because *f*_0,*j*_ will never contribute to *f*_1,*j*_, *f*_0,*j*_ (negative infinite) is always error-free (*f*_0_ in Figure 2E, top-left). Then, we calculate the whole matrix by Equations 6 and 7. Because *f*_0,*j*_ is error-free, *f*_1,*j*_ to $f_{\bar{s},j}$ are correct (*f*_1_ to *f*_4_ in Figure 2E). We save the last register ${\mathrm{MF}}_{\bar{s},j}$:

(9) $${\mathrm{MF}}_{\bar{s},j}=\left[f_{\bar{s},j},f_{\bar{s}*2,j},\ldots ,f_{\bar{s}*(p-1),j}{,f}_{\bar{s}*p,j}\right]$$

Note that we calculate *f*_*i*+1,*j*_ in Equation 7, so **MF**_*S*,*j*_ ([*f*_4,_*f*_8,_*f*_12,_*f*_16_] in Figure 2E) is the last register to store *f* instead of ${\mathrm{MF}}_{\bar{s}\;-\;1,j}$. Because ${\mathrm{MF}}_{\bar{s},j}$ is calculated by ${\mathrm{MF}}_{\bar{s}\;-\;1,j}$_,_ it solves the problems that $f_{Y\;*\;\bar{s}\;+\;\bar{s}\;-\;1,j}$ may update $f_{Y\;*\;\bar{s}\;+\;\bar{s},j}$ (*Y* = 0, 1, 2,…) (Figure S1B). The only exception is that the horizontal gap is long enough to penetrate all the *F* scores $\left(f_{Y\;*\;\bar{s}},f_{Y\;*\;\bar{s}\;+\;1},\ldots ,f_{Y\;*\;\bar{s}\;+\;\bar{s}\;-\;1}\right)$ in the same cell position for all registers (Figure 2C, Figure S1B). If the *F* penetration happens, the value of $f_{x,j}$ is smaller than $f_{x\;-\;\bar{s},j}$ + gap penalty (Figure S1B). Thus, we update the $f_{x,j}$ as the corrected value in advance when we know *F* penetration has happened (correction in Figure 2E). The equation to update all the *f* scores in the first register **MF**_0,*j*_ is following:

(10) $$\begin{array}{c} f_{x,j}=\max\left\{\begin{array}{c} f_{x,j}\\ f_{x\;-\;\bar{s},j}\;+\;\bar{s}\;*\;\mathrm{GapE}\;-\;(H_{x\;-\;1,j\;-\;1}\;-\;H_{x\;-\;1\;-\;\bar{s},j\;-\;1}) \end{array}\right\}\\ x\in (\bar{s},\bar{s}\;*\;2,\ldots ,\bar{s}\;*\;\left(p\;-\;1\right)) \end{array}$$

Now, the updated *f* solves the problems that *f*_*x*−*S*,*j*_ may update *f*_*x*,*j*_. Thus, *f*_Y*S+*S*,*j*_ (*Y* = 1, 2,…) is corrected ([*f*_8_, *f*_12_] in Figure 2). We right shift the last register **MF**_*S*,*j*_*x* bytes (length of *f*_0,*j*_) and update as the first register **MF**_0,*j*_ ([*f*_4,_*f*_8,_*f*_12,_*f*_16_] to [*f*_0,_*f*_4,_*f*_8,_*f*_12_] in Figure 2). After the active F loop, we use the updated *f* as the initial value and recalculate all the values by Equations 6 and 7 (final loop in Figure 2E). Thus, the remaining values are corrected ([*f*_5_, *f*_9_, *f*_13_, *f*_2_, *f*_6_, *f*_10_, *f*_7_, *f*_11_, *f*_15_] in Figure 2, bottom). When all the values in *f* are corrected or error-free, all the values of *H* are corrected.

Specifically, the active F loop and the parallel scan in parasail [18,19] are similar in general. One of the improvements is that the active F loop is implemented in difference recurrence relation, while the parallel scan [18] is implemented in the tradition way, storing and calculating the absolute values. Therefore, the active F loop can increase the number of parallelisms.

### Experimental design

We implemented BSAlign with two modes: “align mode” for pairwise alignment by score matrix, and “edit mode” for pairwise alignment by minimum edit distance. For “align mode”, we compared BSAlign to five programs: SSW (v.1.0) [11], parasail (v.2.4.3) [19], ksw2 (v.current) [15], WFA (v.2.2) [17], and BA (v.0.2.0) [16]. Note that ksw2 implemented the difference recurrence relation [12] and was a component of minimap2 [15]. The scores for the match, mismatch, gap open, and gap extension were set at 2, −4, −4, and −2 for all implementations, respectively. For “edit mode”, we compared BSAlign to Myers (v.myers-agrep) [20] and Edlib (v.1.2.6) [21]. BA was run by Rust WebAssembly (WASM) 128 bits; block size ranged from 32 bp to 2048 bp. Myers’s bit-vector algorithm was one of the fastest deterministic alignment algorithms, but it did not support global alignment and did not trace back the optimal path. Edlib extended Myer’s bit-vector algorithm with additional methods and traced back the optimal path.

We use the same real datasets as BA [16]. The short-read dataset is 100,000 pairs of 101-bp Illumina HiSeq 2000 reads (Accession No. ERX069505). The long-read dataset is 12,477 pairs of around 1000-bp Oxford Nanopore MinION reads (Accession Nos. ERR3278877 to ERR3278886).

We simulated query sequences and reference sequences following a configuration that approximated the error rate in the real dataset for benchmarking. We randomly selected 100 start positions that contained no gap within a 100-kb region from GRCh38. We benchmarked software in three ways: time for length, time and accuracy for divergence and length, and accuracy for long indel and band width. In the “time to length” comparison, we set the reference sizes as 10^2^, 10^2.25^, 10^2.5^, 10^2.75^, 10^3^, 10^3.25^, 10^3.5^, 10^3.75^, 10^4^, 10^4.25^, 10^4.5^, 10^4.75^, and 10^5^ bp with rounding. Then, we used PBSIM2 [22] to simulate a query sequence for each reference region. These query sequences and their reference sequences became pairs of input data in pairwise alignment. Sequences were simulated for both PacBio and Nanopore using hidden Markov models (HMMs) P6C4 and R103. The similarity and mutation ratio (in the format of substitution:insertion:deletion) were set as default values in PBSIM2 (similarity: 85%; mutation ratio: 6:50:54 for PacBio and 23:31:46 for Nanopore). In the “time and accuracy for different divergence and length” comparison, we set the reference sizes as 10^2^, 10^3^, 10^4^, and 10^5^ bp. For each reference size, we also simulated reads with difference divergence (80%, 95%, and 99%). In the “accuracy to long indel and band width” comparison, the reference size is 10^4^ bp and the divergence is 80%. We randomly inserted or deleted sequences of 50, 100, and 200 bp in length in the middle of the reference. For each indel size, we benchmarked software with different band width sizes (32, 64, 128, 256, 512, and 1024 bp).

## Results

We developed the aforementioned algorithms under x86 processors using AVX2 SIMD and tested these programs on a machine with an AMD EPYC 7H12 processor, 1TB random access memory (RAM), and Ubuntu Linux 20.04.1. The execution time was calculated as the sum of user and system time in a single thread. We repeated each alignment experiment 1000 times in repeat mode. To achieve a fair comparison, we modified the implementations to add a repeat mode in SSW [11] and Myers [20]. However, we were unable to add a repeat mode for parasail [19]. We developed a standard Needleman–Wunsch implementation to evaluate the alignment accuracy. As SSW [11] performed local alignment instead of global alignment, we also develop a Smith–Waterman implementation to evaluate SSW’s accuracy. The recall rate was defined as the percentage of alignments that was the same score as the Needleman–Wunsch or Smith–Waterman implementation for global or local alignment, respectively. which may trim the tip sequence and get a higher score in comparison. We also recorded the maximum memory in the system during the software execution. Overall, BSAlign outperformed all the other programs or was on par with the best program in all of the experimental scenarios.

### Evaluation on real data

**Table 1** shows the time and accuracy performance results for five algorithms evaluated using both real and simulated datasets. In the case of processing real datasets, BSAlign maintained 100% recall rate for two datasets. WFA was the fastest algorithm for Illumina reads. In “no band” mode, all algorithms aligned the whole sequences without any band width, and BSAlign was 1.5–6.5 times as fast as ksw2 and SSW for Oxford Nanopore read. In “band” mode, all algorithms can align part of sequences associated with the best alignment according to its method; BA was the fastest algorithm for Oxford Nanopore reads with a recall rate of 87%, and BSAlign was the second fastest algorithm with a recall rate of 100%. Overall, BSAlign is the fastest algorithm with the best recall rate for the Oxford Nanopore dataset.

**Table 1:** Time and accuracy performance of pairwise alignment algorithms

Mode	Algorithm	Real-Illumina		Real-Nanopore		L = 1 kb, d = 5%		L = 10 kb, d = 5%		L = 100 kb, d = 5%		Indel = 50 bp, d = 20%	
		Time (ms)	Recall	Time (ms)	Recall	Time (ms)	Recall	Time (ms)	Recall	Time (ms)	Recall	Time (ms)	Recall
No band	BSAlign	1.65	1.00	3.78	1.00	0.85	1.00	70.00	1.00	3972.13	1.00	46.21	1
	ksw2	1.48	0.98	9.45	1.00	2.22	1.00	207.00	1.00	Error	Error	171.15	1
	SSW	2.71	1.00	24.55	1.00	2.13	1.00	175.00	1.00	2000.22	0.00	121.90	1.00
Band	BSAlign (128 bp)	2.60	1.00	1.92	1.00	0.37	1.00	3.72	1.00	40.70	1.00	2.71	1
	ksw2 (128bp)	1.82	0.98	2.79	1.00	0.58	1.00	5.98	0.78	63.30	0.02	5.01	0
	WFA	0.27	0.99	2.96	0.99	1.18	1.00	11.64	1.00	228.13	1.00	75.8	0.46
	WFA.score	0.12	0.99	1.85	0.99	0.85	1.00	1.18	1.00	50.39	1.00	5.21	0.46
	BA	2.16	0.99	0.91	0.87	0.11	1.00	4.48	1.00	Error	Error	7.57	0

*Note*: WFA.score only computes the alignment score not the complete alignment. BSAlign, Banded Striped Aligner; SSW, Striped Smith–Waterman; WFA, wavefront alignment; BA, block aligner; L, length; d, divergence; indel, insertion and deletion.

### Evaluation of time and accuracy for different lengths

**Figure 3:**
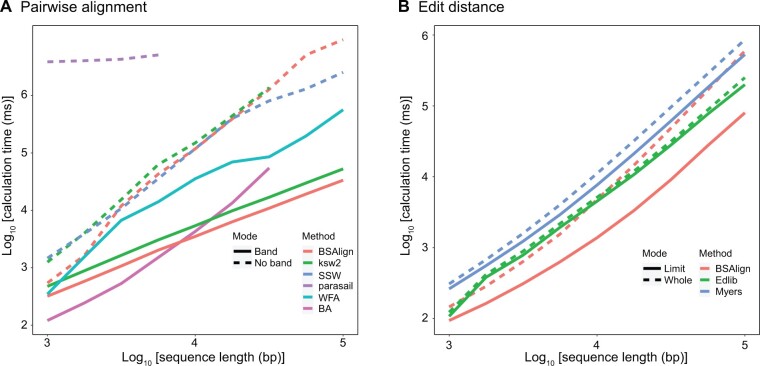
The average computation time for pairwise alignment and edit distance The length of the query sequence pair is from 1000 bp to 100,000 bp. Each pair is run 100 times. **A**. Six implementations: BSAlign, ksw2 [15], SSW [11], parasail [19], WFA [17], and BA [16] are compared. Option band width is set at 128 bp for BSAlign and ksw2. **B**. Three implementations: BSAlign, Edlib [21], and Myers [20] are compared. The mode “whole” and “limit” mean that the maximum edit distance is set at the whole query length and the true edit distance in simulation, respectively. BSAlign, Banded Striped Aligner; SSW, Striped Smith–Waterman; WFA, wavefront alignment; BA, block aligner.

We evaluated all software in three different ways: the running time for different lengths, the running time and accuracy for different divergences and read lengths, and finally the accuracy for different indel sizes and different band widths. In pairwise alignment experiments, BSAlign ran faster than ksw2 [15], SSW [11], and parasail [19] in both “no band” and “band” modes (**Figure 3**A; Table 1). Among all algorithms trailed, only BSAlign and WFA [17] have the capacity to align sequences up to 100 kb in length. BA [16] was at most 3.36 times as fast as BSAlign for 1000-bp sequences. When the sequence length was equal to or longer than 10,000 bp, BSAlign was at most 1.20 to 5.61 times as fast as BA and other algorithms.

### Evaluation for edit distance mode

For the edit distance implementation, BSAlign recorded the fastest speed compared to Myers [20] and Edlib [21] (Figure 3B). The implementations were compared in two modes. In the “whole” mode, all the aligners searched the whole sequences for the minimum edit distance. In the “limit” mode, the minimum edit distance was specified. All the aligners were instructed to stop searching the sequences that were over the minimum edit distance. In the “whole” mode, the fastest implementation switched between BSAlign and Edlib in different sequence lengths. In the “limit” mode, all the aligners ran faster than those in the “whole” mode due to smaller search space, where BSAlign, Myers, and Edlib were 3.10, 1.33, and 1.11 times faster on average, respectively. BSAlign ran 2.13 and 4.32 times as fast as Edlib and Myers on average, respectively. Additionally, BSAlign in edit distance mode is faster than all the pairwise alignment tools (Figure 3A and B).

### Evaluation of time and accuracy for different divergences

Furthermore, we benchmarked this six software for time and accuracy performance under different divergences (**Table 2**). The accuracy of most software was 100%, except for the small size of band width for high-divergence sequences. The time for most software was stable in terms of processing time for different divergences except WFA [17]. Its time for high-divergence sequences (20%) was 4.2 to 16.3 times slower than that of low-divergence sequences. When the length was 1000 bp, WFA and BA were fast and accurate for low-divergence sequences. When the length increased to 10,000 bp, BSAlign (band width 128) was always fastest than other software. When the length further increased to 100,000 bp, BA (capacity overflow), parasail (early termination), and ksw2 (core dumped) collapsed due to memory limitation. BSAlign (band width 128) is reliable and faster than other software.

**Table 2:** Time and accuracy performance for different divergences

Mode	Algorithm	L = 1 kb, d = 1%		L = 1 kb, d = 5%		L = 1 kb, d = 20%		L = 10 kb, d = 1%		L = 10 kb, d = 5%		L = 10 kb, d = 20%		L = 100 kb, d = 1%		L = 100 kb, d = 5%		L = 100 kb, d = 20%	
		Time (ms)	Recall	Time (ms)	Recall	Time (ms)	Recall	Time (ms)	Recall	Time (ms)	Recall	Time (ms)	Recall	Time (ms)	Recall	Time (ms)	Recall	Time (ms)	Recall
No band	BSAlign	0.87	1.00	0.85	1.00	0.76	1.00	70.70	1.00	70.00	1.00	62.50	1.00	91.60	1.00	92.00	1.00	81.10	1.00
	ksw2	2.25	1.00	2.22	1.00	2.01	1.00	209.00	1.00	207.00	1.00	186.00	1.00	Error	Error	Error	Error	Error	Error
	SSW	2.00	1.00	2.13	1.00	2.60	1.00	169.00	1.00	175.00	1.00	197.00	1.00	39.30	0.00	39.30	0.00	52.00	0.00
	parasail	4286.00	1.00	4394.00	1.00	4273.00	1.00	6316.16	1.00	6293.77	1.00	6086.47	1.00	Error	error	Error	Error	Error	Error
Band	BSAlign (128 bp)	0.38	1.00	0.37	1.00	0.37	1.00	3.72	1.00	3.72	1.00	3.64	1.00	40.30	1.00	40.70	1.00	39.80	0.89
	ksw2 (128 bp)	0.57	1.00	0.58	1.00	0.55	1.00	6.06	0.77	5.98	0.74	5.59	0.00	63.70	0.27	63.30	0.02	59.70	0.00
	WFA	0.10	1.00	1.18	1.00	1.63	1.00	8.70	1.00	11.64	1.00	36.84	1.00	181.50	1.00	228.13	1.00	1156.01	1.00
	BA	0.11	1.00	0.11	1.00	0.11	1.00	4.52	1.00	4.48	1.00	4.10	1.00	Error	Error	Error	Error	Error	Error

*Note*: Due to the higher deletion rate in simulation, the total sequence length of 20% divergence is 4.2% and 4.6% shorter than that of 5% and 1% divergences on average, respectively.

### Comparison of indel size, band width, and accuracy

Because the banded methods might miss the optimal path, we further evaluated the influence of indel size on the alignment accuracy (**Table 3**). In this context, we randomly inserted or deleted 50-bp, 100-bp, and 200-bp sequences in the middle of a reference (length = 10 kb, divergence = 20%). Overall, ksw2, SSW, and BSAlign in the “no band” mode were 100% correct. In the “band” mode, WFA detected approximately 50% long indels while BA detected none; ksw2 detected all the indels when the band width was set at 1024 bp; BSAlign with band widths of 512 bp and 1024 bp detected all the 50-bp, 100-bp, and 200-bp indels, respectively. As expected, to accurately detect long indels, the band width should be two times as large as the indel size. It suggests that BSAlign’s striped move is the most accurate strategy to find the optimal path. Regarding the speed, BSAlign was always faster than others. Notably, the lazy F loop implementation SSW [11] ran slower for long indels while the runtime of the active F loop implementation BSAlign was stable for all the indel sizes. It suggests that BSAlign’s active F loop is fast and stable.

**Table 3:** Time and accuracy performance for different indel sizes and band widths

Mode	Algorithm	Indel = 50 bp, d = 20%		Indel = 100 bp, d = 20%		Indel = 200 bp, d = 20%	
		Time (ms)	Recall	Time (ms)	Recall	Time (ms)	Recall
No band	BSAlign	46.21	1.00	45.93	1.00	45.49	1.00
	ksw2	171.15	1.00	171.04	1.00	171.00	1.00
	SSW	121.90	1.00	127.88	1.00	153.19	1.00
Band (128 bp)	BSAlign	2.71	1.00	2.65	0.01	2.64	0.00
	ksw2	5.01	0.00	5.10	0.11	4.90	0.15
Band (256 bp)	BSAlign	3.26	1.00	3.30	1.00	3.22	0.00
	ksw2	9.51	0.43	9.46	0.42	9.28	0.38
Band (512 bp)	BSAlign	4.66	1.00	4.65	1.00	4.62	1.00
	ksw2	18.15	1.00	18.21	0.98	18.03	0.83
Band (1024 bp)	BSAlign	9.03	1.00	9.03	1.00	8.87	1.00
	ksw2	35.16	1.00	35.12	1.00	34.69	1.00
Band	WFA	75.80	0.46	75.08	0.51	76,14	0.61
	BA	7.57	0.00	7.47	0.00	7.48	0.00

### Comparison about memory

We also measured the memory during the execution (**Table 4**). All the software except WFA required similar memory for difference divergences. The memory increased as the sequence lengths increased. Methods in the “no band” mode required a larger memory than those in the “band” mode, as they stored and calculated the whole alignment matrix. For the methods that reducing search space, the banded methods BSAlign and ksw2 required less memory than the other methods (WFA and BA). For example, BSAlign (band width = 128 bp) required 16.36 and 32.46 times less memory than WFA and BA for sequence length of 10 kb and divergence of 20%, respectively.

**Table 4:** Memory for different sequence lengths and divergences

Mode	Algorithm	L = 1 kb			L = 10 kb			L = 100 kb		
		d = 1%	d = 5%	d = 20%	d = 1%	d = 5%	d = 20%	d = 1%	d = 5%	d = 20%
No band	BSAlign	4.19	4.18	3.97	184.72	183.90	162.52	18471.66	18314.93	16433.11
	ksw2	5.16	4.91	4.56	157.56	154.53	43.79	Error	Error	Error
	SSW	4.08	4.43	7.07	10.57	11.82	17.94	24.19	27.56	140.14
Band	BSAlign (128 bp)	2.54	2.52	2.50	5.89	5.88	5.65	40.35	40.18	36.86
	ksw2 (128 bp)	2.13	2.09	2.05	6.80	6.31	7.53	33.14	33.11	31.19
	WFA	8.58	10.39	10.61	37.41	35.00	98.08	153.52	217.70	2095.99
	BA	172.20	175.53	177.19	189.66	189.27	189.04	Error	Error	Error

*Note*: The unit of memory is MB. MB, megabyte.

## Discussion and conclusion

Designing a dynamic banding method is about seeking a balance between speed and accuracy. No software can guarantee both fast and accurate results under any conditions. For example, WFA [17] works well for low-divergence short sequences. BA [16] and ksw2 [15] work well for high-divergence short sequences. When the sequences are short, the band width is short as well. In this case, data structure like striped SIMD is unnecessary and time-wasting. When the sequences are long (10 kb or more), the band width increases as well. In this case, the data structure like striped SIMD is necessary and fast. The striped SIMD banded method BSAlign is faster than the anti-diagonal banding method ksw2 when the band width is 128 bp.

## Code availability

BSAlign is implemented in C programing language and is available at https://github.com/ruanjue/BSAlign.

## CRediT author statement

**Haojing Shao:** Formal Analysis, Investigation, Writing – original draft. **Jue Ruan:** Conceptualization, Methodology, Supervision, Writing – review & editing. Both authors have read and approved the final manuscript.

## Supplementary material

Supplementary material is available at *Genomics, Proteomics & Bioinformatics* online (https://doi.org/10.1093/gpbjnl/qzae025).

## Competing interests

Both authors have declared no competing interests.

## Supplementary Material

qzae025_Supplementary_Data
