# Supplementary material for: BSAlign: A Library for Nucleotide Sequence Alignment
Source: Genomics Proteomics Bioinformatics. 2024 Mar 14;22(2):qzae025. doi: 10.1093/gpbjnl/qzae025 (PMC12016559; doi:10.1093/gpbjnl/qzae025)
Supplement: qzae025_Supplementary_Data [file qzae025_supplementary_data.zip › File S1.docx]

**File S1 Supplementary materials and methods**

**The global alignment of nucleotide sequences**

In the beginning, the algorithm calculates the global alignment by the Needleman-Wunsch algorithm [1]. The two sequences to be aligned, the query sequence and the reference sequence, are defined as Q and R. The length of the query sequence and reference sequence are then defined as Qlen and Rlen, respectively. A matching matrix S(qi, rj) is defined for all residue pairs (a, b) where a,b ∈ {A,T,C,G}. The matching score S(qi,rj) < 0 when qi! = rj and S(qi,rj) > 0 when qi == rj. The penalty for starting a gap and continuing a gap are defined as GapO (gap open, GapO < 0), GapE (gap extension, GapE < 0), and GapOE = GapO + GapE. We keep track of three scoring matrices: E, F, and H, where E represents the alignment score ending with a vertical gap, F represents the alignment score ending with a horizontal gap:

$\left\{ \begin{matrix} E_{i,j}=max\left\{ E_{i,j-1}+GapE,H_{i,j-1}+GapOE \right\} \\ F_{i,j}=max\left\{ F_{i-1,j}+GapE,H_{i-1,j}+GapOE \right\} \\ H_{i,j}=max\left\{ E_{i,j},F_{i,j},H_{i-1,j-1}+S\left( q_{i},r_{j} \right) \right\} \end{matrix} \right\}$ (1)

The cells for Hi,j, Ei,j , and Fi,j are filled by 0 when i<1 or j<1. In our implementation, we store S(qi,rj) in four query profile arrays: S(Q,A), S(Q,C), S(Q,T), and S(Q,G). We calculate the score matrix row by row and extract the S(qi,rj) from query profile column S(Q,rj). We simplify S(qi,rj) as Si,j in this manuscript.

**The striped SIMD data structure**

To accelerate the pairwise alignment in the data structure, we first implemented striped SIMD [2] to the row of the score matrix as well as the query profile arrays. Assuming the query and reference sequences are the row and column in the score matrix, respectively. The row is divided into equal length segments, S. The number of segments, p, is equal to the number of cells being processed in a SIMD register. Take an example in 128 Streaming SIMD Extensions (SSE). When processing byte integers (8-bit values) p = 16 and when processing word integers (16-bit values) p = 8. Hence, p is fixed in the algorithm and S depends on query length (or band width) Qlen: S = ⌈Qlen/p⌉. We first introduced the way to store the non-striped score matrix for each register N in the memory:

N0 =[ H0, H1, H2, ..., Hp−1 ]

N1 = [ Hp, Hp+1, Hp+2, ..., Hp+p−1 ]

...
NS−1 = [ Hp∗(S−1), Hp∗(S−1)+1, Hp∗(S−1)+2, ..., Hp∗(S−1)+p−1 ] (2)

The potential overflow cells in NS−1 are filled by minimum value. In the standard coordinate, there is an inner loop to compute H and F for each register.

After striped conversion, the memory will store the score matrix for each register M :

M0  = [ H0, H0+S, H0+S∗2, ..., H0+S∗(p−1) ]

M1  = [ H1, H1+S, H1+S∗2, ..., H1+S∗(p−1) ]

...

MS−1 = [ HS−1, HS−1+S, HS−1+S∗2, ..., HS−1+S∗(p−1) ] (3)

Hence, the equation to convert each value (Ni,j) in non-striped SIMD(N) to any value (Mi,j) in striped SIMD(M) is:

Ni,j = M(i%S)∗p+⌊i/S⌋,j (4)

In the striped coordinate, all the inner loops to compute H and F are moved outside of the register. Now, Mi+1 depends on Mi. The initial one M0 is solved by the active F loop in the below subsection.

**Edit distance**

Calculating two sequences’ edit distance can be regarded as a special case of pairwise alignment when the mismatch and gap extend are both equal to 1 and, the match and gap open are both equal to 0. Since the difference between adjacent cells belongs to (-1,0,1), the number of bits for storing them is only 2. We can further increase the number of parallelisms using striped SIMD difference recurrence relation. As the number of bits decreases to 2, all the conditions can be enumerated. We converted the difference recurrence relation equation to Boolean logic to further accelerate the calculation.

To simplify the standard pairwise alignment, we only require H, h, u, and v.

ui,j =hi,j −vi−1,j, ∈(−1,0,1)

vi,j = hi,j − ui,j−1, ∈ (−1, 0, 1)

hi,j =min{Si,j,vi−1,j +1,ui,j−1 +1}, ∈(0,1) (5)

To minimize the computation resource, we defined a 2-bit binary code for Boolean logic. For hi,j, ui,j, and vi,j, “-1,0,1” is converted to “10,00,01”. For Si,j, “0,1” is converted to “01,00”. All the conditions for calculating hi,j from Si,j, ui,j−1, and vi−1,j are enumerated as below (Table S1). The new codes are inside the parentheses.

Hence, the Boolean logic for the new $\bar{h}_{i,j}$is following:
$\begin{matrix} \bar{h}_{i,j}^{0}=0 \\ \bar{h}_{i,j}^{1}=\neg(\bar{S}_{i,j}^{1}|\bar{u}_{i,j-1}^{0}|\bar{v}_{i-1,j}^{0}) \end{matrix}$ (6)

All the conditions for calculating ui,j from hi,j and vi−1,j are enumerated as below (Table S2).

As hi,j − vi−1,j ∈ (0, 1), the condition of “hi,j = 1 and vi−1,j = −1” does not exist. Since vi,j is symmetry to ui,j in this definition, the Boolean logic for $\bar{u}_{i,j}$and $\bar{v}_{i,j}$is following:

$\begin{matrix} \bar{u}_{i,j}^{0}=\bar{v}_{i-1,j}^{1}\&({\neg\bar{h}}_{i,j}^{1}) \\ \bar{u}_{i,j}^{1}=\bar{v}_{i-1,j}^{1}\wedge(\bar{h}_{i,j}^{1}|\bar{v}_{i-1,j}^{0}|\bar{v}_{i-1,j}^{1}) \\ \begin{matrix} \bar{v}_{i,j}^{0}=\bar{u}_{i,j-1}^{1}\&({\neg\bar{h}}_{i,j}^{1}) \\ \bar{v}_{i,j}^{1}=\bar{u}_{i-1,j}^{1}\wedge(\bar{h}_{i,j}^{1}|\bar{u}_{i-1,j}^{0}|\bar{u}_{i-1,j}^{1}) \end{matrix} \end{matrix}$ (7)

**References**

[1] Needleman SB, Wunsch CD. A general method applicable to the search for similarities in the amino acid sequence of two proteins. J Mol Biol 1970;48:443–53.

[2] Farrar M. Striped Smith–Waterman speeds database searches six times over other simd implementations. Bioinformatics 2007;23:156–61.
