# Supplementary material for: BSAlign: A Library for Nucleotide Sequence Alignment
Source: Genomics Proteomics Bioinformatics. 2024 Mar 14;22(2):qzae025. doi: 10.1093/gpbjnl/qzae025 (PMC12016559; doi:10.1093/gpbjnl/qzae025)
Supplement: qzae025_Supplementary_Data [file qzae025_supplementary_data.zip › Supplementary material captions.docx]

**Supplementary material**

**File S1 Supplementary materials and methods**

**Figure S1 The F loop in non-striped order A–C**

The three panels show how the pairwise alignment calculates a cell (h12,j) in non-striped order in three situations: (**A**) no gap, (**B**) short horizontal gap and (**C**) long horizontal gap. The dash line indicates the optimal path.

**Table S1 In edit distance mode, enumeration of conditions for converting hi,j from Si,j, ui,j−1 and vi−1,j**

**Table S2 In edit distance mode, enumeration of conditions for converting ui,j from hi,j and vi−1,j**
