# Supplementary material for: BSAlign: A Library for Nucleotide Sequence Alignment
Source: Genomics Proteomics Bioinformatics. 2024 Mar 14;22(2):qzae025. doi: 10.1093/gpbjnl/qzae025 (PMC12016559; doi:10.1093/gpbjnl/qzae025)
Supplement: qzae025_Supplementary_Data [file qzae025_supplementary_data.zip › Table S1.docx]

**Table S1 In edit distance mode, enumeration of conditions for converting hi,j from Si,j, ui,j−1 and vi−1,j**

| $S_{i,j}(\bar{S}_{i,j}^{0}\bar{S}_{i,j}^{1})$ | $u_{i,j-1}(\bar{u}_{i,j-1}^{0}\bar{u}_{i,j-1}^{1})$ | $v_{i-1,j}(\bar{v}_{i-1,j}^{0}\bar{v}_{i-1,j}^{1})$ | = | $h_{i,j}(\bar{h}_{i,j}^{0}\bar{h}_{i,j}^{1})$ |
| --- | --- | --- | --- | --- |
| 0(01) | -1(10) | -1(10) | = | 0(00) |
| 0(01) | -1(10) | 0(00) | = | 0(00) |
| 0(01) | -1(10) | 1(01) | = | 0(00) |
| 0(01) | 0(00) | -1(10) | = | 0(00) |
| 0(01) | 0(00) | 0(00) | = | 0(00) |
| 0(01) | 0(00) | 1(01) | = | 0(00) |
| 0(01) | 1(01) | -1(10) | = | 0(00) |
| 0(01) | 1(01) | 0(00) | = | 0(00) |
| 0(01) | 1(01) | 1(01) | = | 0(00) |
| 1(00) | -1(10) | -1(10) | = | 0(00) |
| 1(00) | -1(10) | 0(00) | = | 0(00) |
| 1(00) | -1(10) | 1(01) | = | 0(00) |
| 1(00) | 0(00) | -1(10) | = | 0(00) |
| 1(00) | 0(00) | 0(00) | = | 1(01) |
| 1(00) | 0(00) | 1(01) | = | 1(01) |
| 1(00) | 1(01) | -1(10) | = | 0(00) |
| 1(00) | 1(01) | 0(00) | = | 1(01) |
| 1(00) | 1(01) | 1(01) | = | 1(01) |

*Note*: The new binary codes are inside the parentheses.
