# Supplementary material for: BSAlign: A Library for Nucleotide Sequence Alignment
Source: Genomics Proteomics Bioinformatics. 2024 Mar 14;22(2):qzae025. doi: 10.1093/gpbjnl/qzae025 (PMC12016559; doi:10.1093/gpbjnl/qzae025)
Supplement: qzae025_Supplementary_Data [file qzae025_supplementary_data.zip › Table S2.docx]

**Table S2 In edit distance mode, enumeration of conditions for converting ui,j from hi,j and vi−1,j**

| $\boldsymbol{h}_{\boldsymbol{i},\boldsymbol{j}}({\bar{\boldsymbol{h}}}_{\boldsymbol{i},\boldsymbol{j}}^{\mathbf{0}}{\bar{\boldsymbol{h}}}_{\boldsymbol{i},\boldsymbol{j}}^{\mathbf{1}})$ | $\boldsymbol{v}_{\boldsymbol{i}-\mathbf{1},\boldsymbol{j}}({\bar{\boldsymbol{v}}}_{\boldsymbol{i}-\mathbf{1},\boldsymbol{j}}^{\mathbf{0}}{\bar{\boldsymbol{v}}}_{\boldsymbol{i}-\mathbf{1},\boldsymbol{j}}^{\mathbf{1}})$ | = | $u_{\boldsymbol{i},\boldsymbol{j}}(\bar{u}_{\boldsymbol{i},\boldsymbol{j}}^{\mathbf{0}}\bar{u}_{\boldsymbol{i},\boldsymbol{j}}^{\mathbf{1}})$ |
| --- | --- | --- | --- |
| 0(00) | 0(00) | = | 0(00) |
| 0(00) | 1(01) | = | -1(10) |
| 0(00) | -1(10) | = | 1(01) |
| 1(01) | 0(00) | = | 1(01) |
| 1(01) | 1(01) | = | 0(00) |

*Note*: The new binary codes are inside the parentheses.
