# Supplementary material for: BSAlign: A Library for Nucleotide Sequence Alignment
Source: Genomics Proteomics Bioinformatics. 2024 Mar 14;22(2):qzae025. doi: 10.1093/gpbjnl/qzae025 (PMC12016559; doi:10.1093/gpbjnl/qzae025)
Supplement: qzae025_Supplementary_Data [file qzae025_supplementary_data.zip › FigureS1.pdf]

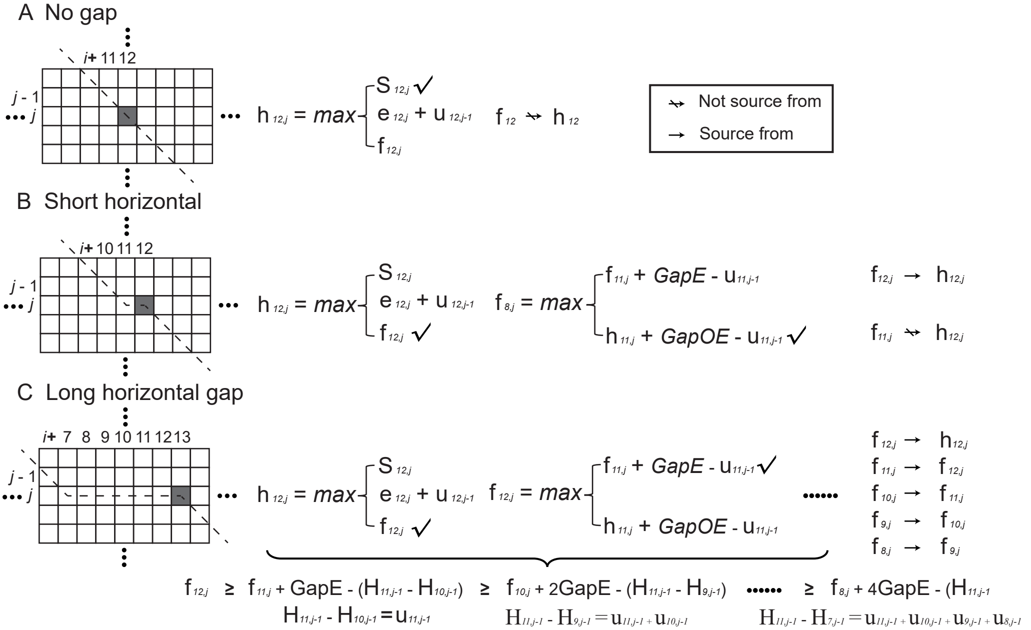
A No gap
i+ 11 12
j - 1
j
h 12,j = max { S 12,j ✓ ; e 12,j + u 12,j-1 ; f 12,j }
f 12 ↛ h 12
↛ Not source from
→ Source from
B Short horizontal
i+ 10 11 12
j - 1
j
h 12,j = max { S 12,j ; e 12,j + u 12,j-1 ; f 12,j ✓ }
f 8,j = max { f 11,j + GapE - u 11,j-1 ; h 11,j + GapOE - u 11,j-1 ✓ }
f 12,j → h 12,j
f 11,j ↛ h 12,j
C Long horizontal gap
i+ 7 8 9 10 11 12 13
j - 1
j
h 12,j = max { S 12,j ; e 12,j + u 12,j-1 ; f 12,j ✓ }
f 12,j = max { f 11,j + GapE - u 11,j-1 ✓ ; h 11,j + GapOE - u 11,j-1 }
......
f 12,j → h 12,j
f 11,j → f 12,j
f 10,j → f 11,j
f 9,j → f 10,j
f 8,j → f 9,j
f 12,j ≥ f 11,j + GapE - (H 11,j-1 - H 10,j-1) ≥ f 10,j + 2GapE - (H 11,j-1 - H 9,j-1) ...... ≥ f 8,j + 4GapE - (H 11,j-1
H 11,j-1 - H 10,j-1 = u 11,j-1
H 11,j-1 - H 9,j-1 = u 11,j-1 + u 10,j-1
H 11,j-1 - H 7,j-1 = u 11,j-1 + u 10,j-1 + u 9,j-1 + u 8,j-1
